# The opioid epidemic among the Latino population in California

**DOI:** 10.1016/j.dadr.2022.100029

**Published:** 2022-02-02

**Authors:** Avelardo Valdez, Alice Cepeda, Jessica Frankeberger, Kathryn M. Nowotny

**Affiliations:** a Suzanne Dworak-Peck School of Social Work, University of Southern California, Montgomery Ross Fisher Building, 669 W. 34th St., Los Angeles, CA 90089, USA; b Department of Behavioral and Community Health Sciences, University of Pittsburgh Graduate School of Public Health, 130 De Soto Street, Pittsburgh, PA 15261, USA; c Department of Sociology, University of Miami, 5202 University Dr., Merrick Building 120, Coral Gables, FL 33146, USA

**Keywords:** Opioids, Heroin, Fentanyl, Overdose, Hispanic/Latino, Mexican-origin populations

## Abstract

**Background:**

The opioid epidemic in the United States has manifested differently across geographic regions and populations, with recent increases among racial/ethnic minorities and in the Western region of the U.S. This study provides an overview of the opioid overdose epidemic among Latinos in California and highlights high-risk areas in the state.

**Methods:**

Using publicly available data from California, we examined trends in opioid-related deaths (e.g., overdose) and opioid-related emergency department (ED) visits among Latinos at the county-level, as well as changes in opioid outcomes overtime.

**Results:**

Opioid-related death rates among Latinos (mostly Mexican-origin) in California remained relatively stable from 2006–2016, but started to increase in 2017 peaking at an age-adjusted opioid mortality rate of 5.4 deaths per 100,000 Latino residents in 2019. Prescription opioid-related deaths, compared to heroin and fentanyl, have remained the highest over time. However, fentanyl-related deaths began to increase dramatically in 2015. Lassen, Lake, and San Francisco counties had the highest 2019 opioid-related death rates among Latinos. Opioid-related ED visits among Latinos have steadily increased since 2006 with a sharp increase in rates in 2019. San Francisco, Amador, and Imperial counties had the highest 2019 rates of ED visits.

**Conclusions:**

Latinos are facing detrimental consequences associated with recent increasing trends in opioid overdoses. The identified high-risk counties may have vulnerable sub-populations of Latinos, such as those in northern rural regions, that have gone underrepresented in conventional surveillance health databases. Time sensitive policies and interventions are needed to curtail health consequences especially among “hidden” Latino populations.

## Highlights

• In California, opioid mortality rates among Latinos remained stable from 2006–2016.
• Starting in 2017, opioid-related death rates increased dramatically among Latinos.
• Rates of opioid-related ED visits steadily increased in this population since 2006.
• High-risk counties for opioid-related deaths and ED visits are identified.

## 1 Introduction

The opioid epidemic has manifested differently across U.S. geographic regions, with fatal opioid overdose rates ranging from 4 to 42 per 100,000 residents (National Institute on Drug Abuse, 2020b). Yet, much of the research has focused on white, rural, and male populations in the Midwest and East Coast (Dombrowski et al., 2016, McKnight and Des Jarlais, 2018, McLean, 2016, Rouhani et al., 2020). Less known is the geographic variation in the opioid epidemic in other parts of the US. For instance, opioid-related deaths have increased in the Western region of the US in recent years (Ciccarone, 2021, Shover et al., 2020), particularly fentanyl-related deaths, which increased more than 60% from 2017 to 2018 in California (National Institute on Drug Abuse, 2020a). While state-level indicators provide one segment of the story, further analyses are needed to identify potentially vulnerable groups of people who use opioids within subregions of the U.S. This is particularly relevant given the ever-changing nature of the epidemic Ciccarone (2021).

Recent attention has been given to the emerging national trends in increased opioid use among racial/ethnic minorities Cano (2021). While heroin-related mortality rates have decreased among non-Latino Whites since 2018, increases have been observed among non-Latino Blacks and Latinos (Drake et al., 2020, Shiels et al., 2018). Among Latinos, opioid-related death rates have almost doubled from 1999 to 2017, with particular increases in synthetic opioid-related deaths (Cano, 2020, Drake et al., 2020). In 2017, Fentanyl was most commonly involved in overdose deaths among Latinos (40.2%), followed by heroin (31.2%) and cocaine (26.8%) (Cano, 2020). Of significance, Cano (2020) has found that there exists substantial variation in Latino drug overdose mortality rates that may be concealing high-risk subgroups. For instance, findings point that while California and Texas had some of the lowest Hispanic drug overdose mortality rates, they still had the highest prevalence of Hispanic overdoses, representing more than a quarter of all Hispanic deaths in the U.S. between 2014–2017.

Building upon this existing literature, this study utilizes data from California to examine within state geographic variation in opioid overdoses among Latinos. California not only has the largest Latino population in the country, encompassing nearly 40% of the state and a large immigrant population (37% of Latinos in California are foreign-born), but is also overwhelmingly home to Mexican-origin Latinos, which represent the largest subgroup in the nation (U.S. Census Bureau, 2019). The social ecology of the state contains a diversity of large urban regions, 21 rural counties, large agriculture and migrant labor sectors, and numerous areas with limited or inequitable health resources, including drug treatment and harm reduction access (Langabeer et al., 2020, Rocha, 2017, Sharma et al., 2017). Thus, with Latinos making up a growing proportion of the US, California provides a unique opportunity to understand the nature and extent of opioid overdoses for Latino subgroups living in distinct contexts across the state. First, we examine rates of opioid-related mortality among Latinos in California from 2006 to 2019 by type of opioid. Second, we document the incidence of opioid-related deaths and opioid-related emergency department visits at the county-level for Latinos in California from 2006–2019.

## 2 Materials and methods

Data were publicly available from the State of California. The unit of analysis is the county (n=58). Eight counties are considered urban, eight suburban, 21 mid-size/small, and 21 rural using US Census Bureau designations. This research is not considered human subjects and was exempt from IRB review.

The two outcomes are opioid-related deaths (i.e., fatal overdoses) and opioid-related emergency department (ED) visits (i.e., nonfatal overdoses) among Latinos. Both variables were obtained from the California Opioid Overdose Surveillance Dashboard for 2006–2019 (California Department of Public Health, 2020). Opioid-related deaths are examined for all opioids, prescription opioids, heroin, and fentanyl. Deaths were identified based on vital statistics in the California Comprehensive Death File and were classified using ICD-10-CM codes as the underlying cause of death: X40-X44, X60-X64, X85, or Y10-Y14; and with the ICD-10-CM multiple causes of death codes: T40.0-T40.4 or T40.6 for opium, heroin, natural and semisynthetic opioids, methadone, synthetic opioids and other and unspecified narcotics. Opioid-related ED visits reflect non-fatal acute poisonings due to the effects of opioids regardless of intent. ED visits due to late/adverse effects and chronic poisonings (e.g., damage to organs from long-term use) are excluded. ED visits that resulted in a fatal overdose are excluded. These data are collected by the California Office of Statewide Health Planning and Development and use the same ICD-10-CM codes for principal diagnosis of opioid poisonings. County population statistics for 2019 were obtained from the U.S. Census Bureau (U.S. Census Bureau, 2019).

Analyses were descriptive and were completed using Microsoft Excel. All outcomes are presented as age-adjusted rates per 100,000 at the county-level. Age adjustments were made using direct standardization to the US standard population. We first compare race/ethnicity-specific age-adjusted incidence of opioid mortality. Then, we examine Latino-specific age-adjusted rates for the two opioid outcomes (i.e., deaths and ED visits) overtime across counties.

## 3 Results

### 3.1 Latino opioid-related deaths

Opioid-related deaths among Latinos in California remained relatively stable from 2006–2016 with an average age-adjusted rate of 2.6 deaths (per 100,000) annually. In 2017, opioid mortality started to increase, reaching an age-adjusted opioid-related death rate of 5.4 per 100,000 in 2019. This 2019 age-adjusted opioid-related overdose death rate was well below the national rate for Latinos (12.7 per 100,000 people) (Minino and Hedegaard, 2021). Within the state, Latinos have consistently had some of the lowest overdose rates among all racial/ethnic groups. In 2019, the age-adjusted opioid-related death rate among Latinos was lower than non-Latino Whites (12.6), Blacks (12.3), and Native Americans (15.7), but higher than Asians (1.4).

**Fig. 1:**
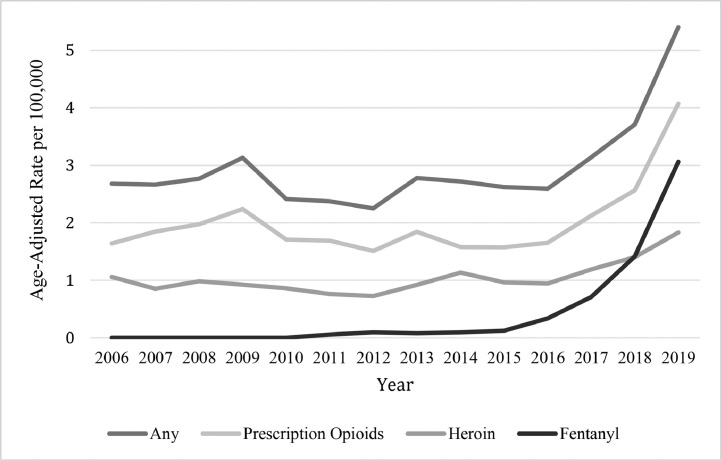
Opioid-related deaths among Latinos in California, 2006–2019 Fig. 1 depicts the trends in age-adjusted rates of opioid-related mortality among Latinos in California from 2006 to 2019 by type of opioid.

Deaths specifically related to prescription opioids among Latinos have remained the highest over time, averaging 2.0 deaths per 100,000 and increasing to 4.1 by 2019 (Fig. 1). In 2019, heroin and fentanyl-related deaths increased among Latinos by 1.3 and 2.2 times their 2018 rates, respectively. The age-adjusted fentanyl-related death rate more than doubled from 1.4 in 2018 to 3.1 in 2019, surpassing that of heroin-related deaths of 1.8 deaths per 100,000. Despite statewide lower rates, Latinos in California are a heterogeneous population residing in distinct geographic contexts that warrant a closer examination.

**Fig. 2:**
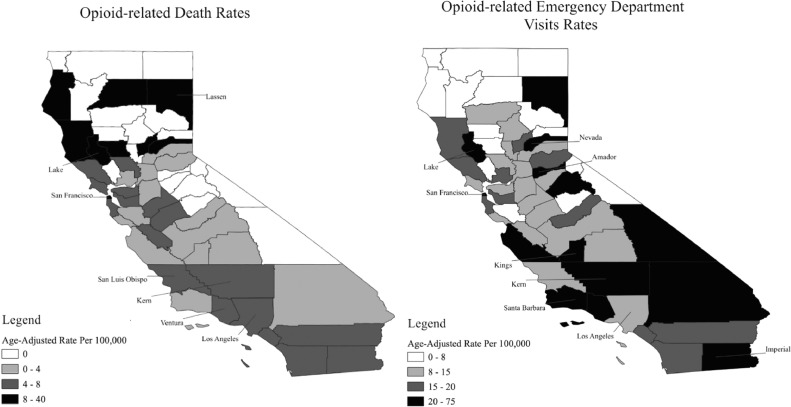
Rates of opioid-related deaths and emergency department visits among Latinos in California, 2019 Fig. 2 depicts the age-adjusted rates of opioid-related deaths (left panel) and emergency department visits (right panel) among Latinos in California counties in 2019. Labels of county names are included for those counties described in the text.

Overall, the northern region of California represents the highest levels of opioid-related deaths among Latinos. In 2019, Lassen, Lake, and San Francisco counties had the highest opioid-related mortality rates in the state with all three counties having rates over five times the state age-adjusted rate of 5.4 per 100,000. In Lassen County, age-adjusted opioid death rates among Latinos have increased from 20.1 in 2006 to 39.5 in 2019, a 96% increase. In Lake County, opioid mortality rates ranged from 8.7 per 100,000 people in 2006 to 32.6 in 2019. In San Francisco County, opioid-related deaths increased by over 150% in the last decade reaching 28.3 per 100,000 in 2019. It should be noted that there are a few counties in central and southern California, such as San Luis Obispo (7.8), Ventura (7.7) and Kern (7.5), that reflect elevated trends. Comparatively, the urban county of Los Angeles had Latino opioid-death rates in 2018 and 2019 lower than the state average.

### 3.2 Latino opioid-related emergency department (ED) visits

Age-adjusted rates of opioid-related ED visits among Latinos steadily increased from 7.6 per 100,000 in 2006 to 14.0 in 2018. In 2019, ED visits among Latinos increased dramatically to 18.9 and preliminary data suggests this continued into 2020. Heroin-related ED visits among Latinos remained relatively stable from 2006 through 2014 but increased starting in 2015. By 2019, the heroin-related ED visit rate increased to 7.7, a 108% increase from 2006. ED visit rates related to prescription opioids or fentanyl were not available.

The county with the highest age-adjusted rate of opioid ED visits among Latinos in 2019 was San Francisco (74.8 per 100,000), followed distantly by Amador (42.3) and Imperial (42.2). Despite the latter two counties having some of the highest ED rates in the state, they do not have high opioid-related death rates. For example, Amador had no opioid-related deaths among Latinos in 2019. In contrast, Lake County, which had the second highest Latino opioid mortality rate (32.6), had the fourth highest ED visit rate (40.0) in the state. Additional counties across central and northern California, such as Nevada (37.2), Kings (30.4), Santa Barbara (27.5), and Kern (27.1), also report high ED visit patterns among Latinos. Moreover, with the exception of San Francisco, most California urban counties had opioid-related ED visit rates similar or lower than the state average. For example, Latinos in Los Angeles County had a 2019 age-adjusted opioid-related ED visit rate of 11.2 per 100,000, compared to 18.9 across the state.

## 4 Discussion

While opioid overdose rates in California have remained relatively low and stable since 2006, there have been notable increases in age-adjusted opioid-related death and ED visit rates among Latinos since 2017. Specifically, they are facing spikes in fentanyl-related overdoses and an increasing trends in prescription opioid and heroin overdoses. Moreover, opioid-related ED visits among Latinos have more than doubled since 2006. These overall state-level patterns suggest the need for time-sensitive policies and preventative interventions to curtail existing and future health disparities associated with undetected increases in opioid use patterns in the Latino community. These interventions, however, cannot be a one-size fit all approach and require more tailorized approaches that can only come from subregion and subgroup patterns.

County-level results from this study highlight the diverse nature of fatal and non-fatal opioid overdose patterns for Latinos living in distinct regions of California. Findings indicate that state-level rates conceal high-risk subgroups of Latinos living in specific geographic contexts. Latinos in poor and rural Northern California counties are disproportionately experiencing higher rates of opioid-related outcomes. Counties such as Lassen, Lake and Amador have a Latino population that ranges between 15–22%, with an average annual income of $55,000 and less than 10% foreign-born. These counties are disproportionally representative of agricultural and husbandry-based economies with potentially large workforces of Mexican-origin immigrants (Flores, 2016). Unlike those in urban counties, Latinos in these communities may be living in socially isolated and economically marginalized contexts with less access to culturally tailored healthcare, drug treatment, and harm reduction services (Negi et al., 2021, Stone et al., 2019). For instance, all three counties mentioned above report having no methadone clinics, and only Lake county has a syringe exchange program (California Department of Public Health, 2021, Substance Abuse and Mental Health Services Administrtation, 2021). Given these characteristics and the high rates of prescription opioid-related deaths, future research should focus on work-related injuries as possible precursors for the misuse of opioids for pain management among Latinos. Overall, Latinos living in poor, rural and isolated counties with similar profiles spanning across California (Venture, Kern, Imperial) and other regions of the U.S. (with emerging Latino populations) may benefit from closer monitoring and prevention efforts to reduce opioid-related overdoses.

Latinos living in large urban cities like San Francisco represent a distinct profile from that of the rural counties previously described. Unlike other urban or suburban counties, San Francisco had some of the highest rates of opioid-related deaths and ED visits for Latinos over the last decade. Several considerations should be noted for opioid-related patterns in this county. First, San Francisco is a highly urbanized county where access to services is more widely available, unlike other counties like Los Angeles which includes a mix of large dense cities, suburbs, small municipalities, and rural areas. Despite this, San Francisco and the Bay Area have the highest levels of income disparity in the state Bohn and Thorman, 2020, contributing to a large and vulnerable homeless population at disproportionate risk for overdoses in the city, of which 18% are Latino (Applied Survey Research, 2019). Additionally, death rates in San Francisco may be partially explained given the relatively small percentage of Latinos living here compared to Southern California (15% vs. 49% in Los Angeles). With nearly 40% of the population foreign-born, Latinos in San Francisco may face additional barriers to accessing culturally appropriate (e.g., language barriers) health and drug treatment services. Overall, research is further needed to determine the impact these types of barriers and factors, such as MAT, cannabis-related policy, and other considerations, are having in urban geographic contexts across the country.

### 4.1 Limitations

While this study has begun to disentangle temporal and geographic trends in Latino opioid overdoses, limitations should be considered. We used public administrative data, which may be suspectable to misclassification. Opioid-related death and ED visit outcomes also do not distinguish between unintentional or intentional (self-harm) overdose and should be cautiously interpreted. All outcomes are presented at the county-level. While informative, there may be overlooked local spatial patterning and individual-level factors that contribute to opioid overdose among this population. Although the present analyses do not focus on racial/ethnic comparisons, it should be noted that Lassen is one of the few counties where opioid related outcomes are uniquely impacting Latinos, compared to Whites and Blacks. Additional analyses are needed at the county level to further disentangle racial/ethnic group differences (Cano, 2021).

Lastly, this study focuses solely on trends among Latinos in California, which may not be generalizable to Latino populations in other regions of the U.S. For instance, the majority of the California Latino population are Mexican-origin and there exists a large foreign-born population (U.S. Census Bureau, 2019a). Despite this, California may be one of the few states where emerging opioid trends can be examined among the Latino population living in distinct geographic settings across the country. For instance, California reflects diverse immigration settlement patterns (urban vs rural), drug markets, and drug policy (e.g., medical and recreational cannabis laws) that may be obscuring the impact opioid overdoses are having in this population (Cano, 2020). Nevertheless, further research in other regions of the U.S. is warranted.

### 4.2 Conclusions

We have provided an initial overview and portrait of the patterns, trends, and rates of opioid overdoses among Latinos in California. Research should continue to monitor these trends and identify modifiable risk factors and contexts to be targeted for interventions. Particular attention should be given to identifying and addressing barriers (such as stigma) that prevent Latinos from seeking medical, social, and drug treatment services at state and county levels, particularly among vulnerable populations such as immigrants and agricultural workers. Policymakers should consider cultural factors in providing harm reduction services to mitigate the consequences of opioid use among this population. Research is needed to understand how distinct contexts, including healthcare and treatment access, impact the California Latino population and other Latinos in similar contexts across the country that may be facing disproportionate opioid overdose risks .

## 5 Author disclosures

### 5.1 Role of funding source

This study was supported by the Drug Policy Alliance of California. The funders had no role in the study design, collection, analysis or interpretation of the data, writing the manuscript, or decision to submit the paper for publication.

## 6 Contributions

AV and AC conceptualized the study design and analysis and led the writing of the manuscript. KM and JF conducted the analysis and contributed to the interpretation, writing, and editing of the manuscript. All authors approved the final manuscript.

## Declaration of Competing Interest

The authors declare that they have no known competing financial interests or personal relationships that could have appeared to influence the work reported in this paper.
